# Over-expressing the soluble gp130-Fc does not ameliorate methionine and choline deficient diet-induced non alcoholic steatohepatitis in mice

**DOI:** 10.1371/journal.pone.0179099

**Published:** 2017-06-20

**Authors:** Helene L. Kammoun, Tamara Louise Allen, Darren Colin Henstridge, Michael James Kraakman, Lone Peijs, Stefan Rose-John, Mark Anthony Febbraio

**Affiliations:** 1 Cellular and Molecular Metabolism Laboratory, Baker Heart & Diabetes Institute, Melbourne, Australia; 2 Immunology department, Monash University, Melbourne, Australia; 3 Department of Biochemistry, Christian-Albrechts-Universität zu Kiel, Kiel, Germany; 4 Cellular and Molecular Metabolism, Garvan Institute, Sydney, Australia; University of Navarra School of Medicine and Center for Applied Medical Research (CIMA), SPAIN

## Abstract

Non-alcoholic steatohepatitis (NASH) is a liver disease with the potential to lead to cirrhosis and hepatocellular carcinoma. Interleukin-6 (IL-6) has been implicated in the pathogenesis of NASH, with the so-called IL-6 ‘trans-signaling’ cascade being responsible for the pro-inflammatory actions of this cytokine. We aimed to block IL-6 ‘trans-signaling’, using a transgenic mouse that overexpresses human soluble glycoprotein130 (sgp130Fc Tg mice) fed a commonly used dietary model of inducing NASH (methionine and choline deficient-diet; MCD diet) and hypothesized that markers of NASH would be ameliorated in such mice. Sgp130Fc Tg and littermate control mice were fed a MCD or control diet for 4 weeks. The MCD diet induced many hallmarks of NASH including hepatomegaly, steatosis, and liver inflammation. However, in contrast with other mouse models and, indeed, human NASH, the MCD diet model did not increase the mRNA or protein expression of IL-6. Not surprisingly, therefore, markers of MCD diet-induced NASH were unaffected by sgp130Fc transgenic expression. While the MCD diet model induces many pathophysiological markers of NASH, it does not induce increased IL-6 expression in the liver, a key hallmark of human NASH. We, therefore, caution the use of the MCD diet as a viable mouse model of NASH.

## Introduction

Non-alcoholic fatty liver disease (NAFLD) is the most common form of chronic liver disease representing a significant burden on healthcare systems. Non-alcoholic fatty liver disease (NAFLD) represents the hepatic component of the metabolic syndrome and has been following the same epidemic rate as obesity [1]. NAFLD covers a spectrum of metabolic liver diseases from the simple fatty liver to life threatening conditions such as cirrhosis and even hepatocellular carcinoma (HCC). Non-alcoholic steatoheopatitis (NASH) is a turning point of the pathogenesis of NAFLD as it is the first step of the disease that is irreversible [2]. NASH consists of hepatic steatosis accompanied by cellular ballooning, inflammation and fibrosis [2]. The pro-inflammatory cytokine interleukin-6 (IL-6) has been implicated in the pathogenesis and development of NASH in rodent models and, indeed, in humans [3–8]. Paradoxically, work from our laboratory and others have highlighted that complete ablation of IL-6 in mice can lead to deleterious metabolic consequences such as glucose intolerance, obesity and more importantly increased liver steatosis and damage [9–11]. Consequently, this would in fact render the use of complete IL-6 blockade, currently a therapy for diseases such as Rheumatoid Arthritis [12], a poor therapeutic option for NASH. The controversy regarding the role of IL-6 in metabolic homeostasis may be due to its complex signaling biology [13]. IL-6 can signal in two distinct manners referred to as ‘classical signaling’ and ‘trans-signaling’. IL-6 classical signaling, thought to mediate the positive metabolic effect of the cytokine, occurs when IL-6 binds to the membrane IL-6 receptor (IL-6R) and signals through homodimerization of two glycoprotein130 receptors (gp130R). Numerous cell types, including hepatocytes, endothelial cells and most immune cells express IL-6R but all cells express the gp130R. IL-6 trans-signaling is a process by which the IL-6R can be cleaved by metalloproteinases, resulting in a soluble form of the IL-6R (sIL-6R). IL-6 can bind to the sIL-6R, giving this circulating ligand/receptor complex the capacity to signal in cells that express the gp130R, but not IL-6R, on their plasma membrane [14]. Importantly, work from our group has shown that blocking IL-6 trans-signaling using a mouse model that overexpresses a recombinant soluble form of the gp130 (sgp130Fc), can prevent inflammation in an air-pouch model of local inflammation [14], in atherosclerotic lesions [15] and adipose tissue beds during high fat feeding [16]. Notably, sgp130Fc does not block the activity of IL-6 mediated signaling via the membrane bound IL-6R [17]. Accordingly, we hypothesized that sgp130Fc transgenic mice would be at least partially protected from NASH without exhibiting the deleterious hepatic effects observed by complete ablation of IL-6.

## Results

### Overexpression of sgp130Fc does not prevent reduced fasting glucose or weight loss induced by the MCD diet

**Fig 1:**
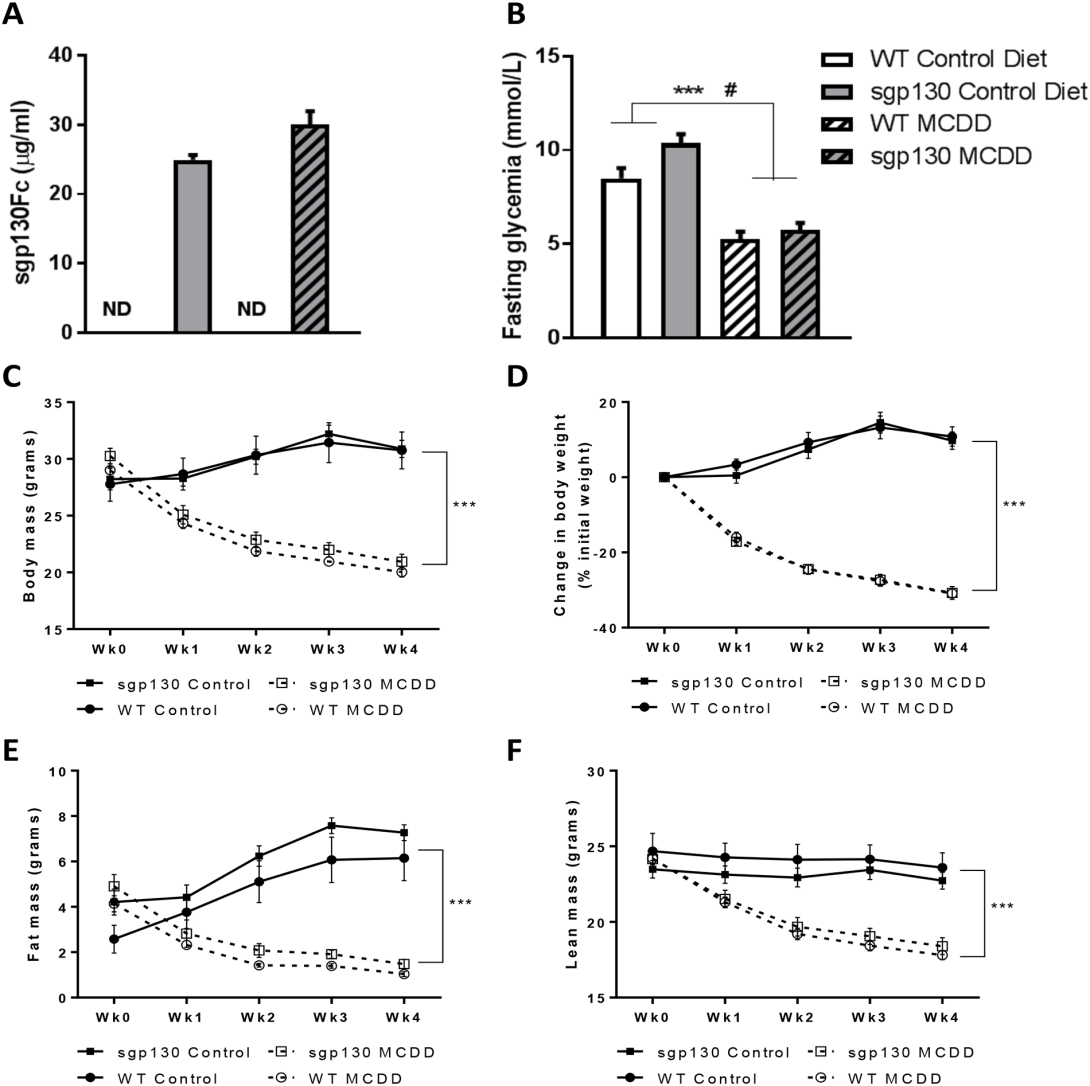
Overexpression of sgp130Fc does not impact MCD diet induced weight loss. (A) Concentration of soluble gp130 Fc (sgp130Fc) transgene in plasma of mice at 3 weeks of age (B) Fasting glycemia after 4 weeks of methionine and choline deficient diet (MCDD). (C) Body mass, (D) weight change expressed as percent initial body weight (E) EchoMRI fat mass and (F) EchoMRI lean mass in control and MCDD mice over the course of dietary intervention. Data expressed as average ± SEM, n = 6–7. Statistical analysis by 2 way ANOVA, ***p<0.001 Main effect for Control vs MCDD; # p<0.05 Main effect for WT vs sgp130Fc Tg.

The circulating sgp130Fc levels ranged between 20–30 μg/ml in the sgp130Fc transgenic mice and were undetected in WT mice, irrespective of diet (Fig 1A). The MCD diet is commonly associated with reduced fasting glycemia and weight loss (up to 25% of initial body weight), due to increased β oxidation in parallel with lipid accumulation arising from the VLDL secretion inhibition [18]. Accordingly, we observed a marked decrease in fasting glucose, body mass, fat mass and lean mass in all MCD diet fed mice (Fig 1B–1F). Of note, however, there were no differences in any of the body composition measures when comparing sgp130Fc with WT mice (Fig 1C–1F). Interestingly, statistical analysis of the fasting glycemia showed a main genotype effect in addition to the diet effect which might be linked with the trending increase in fat mass in the transgenic animals on Control diet.

### Overexpression of sgp130Fc does not prevent MCD diet induced dyslipidemia

**Fig 2:**
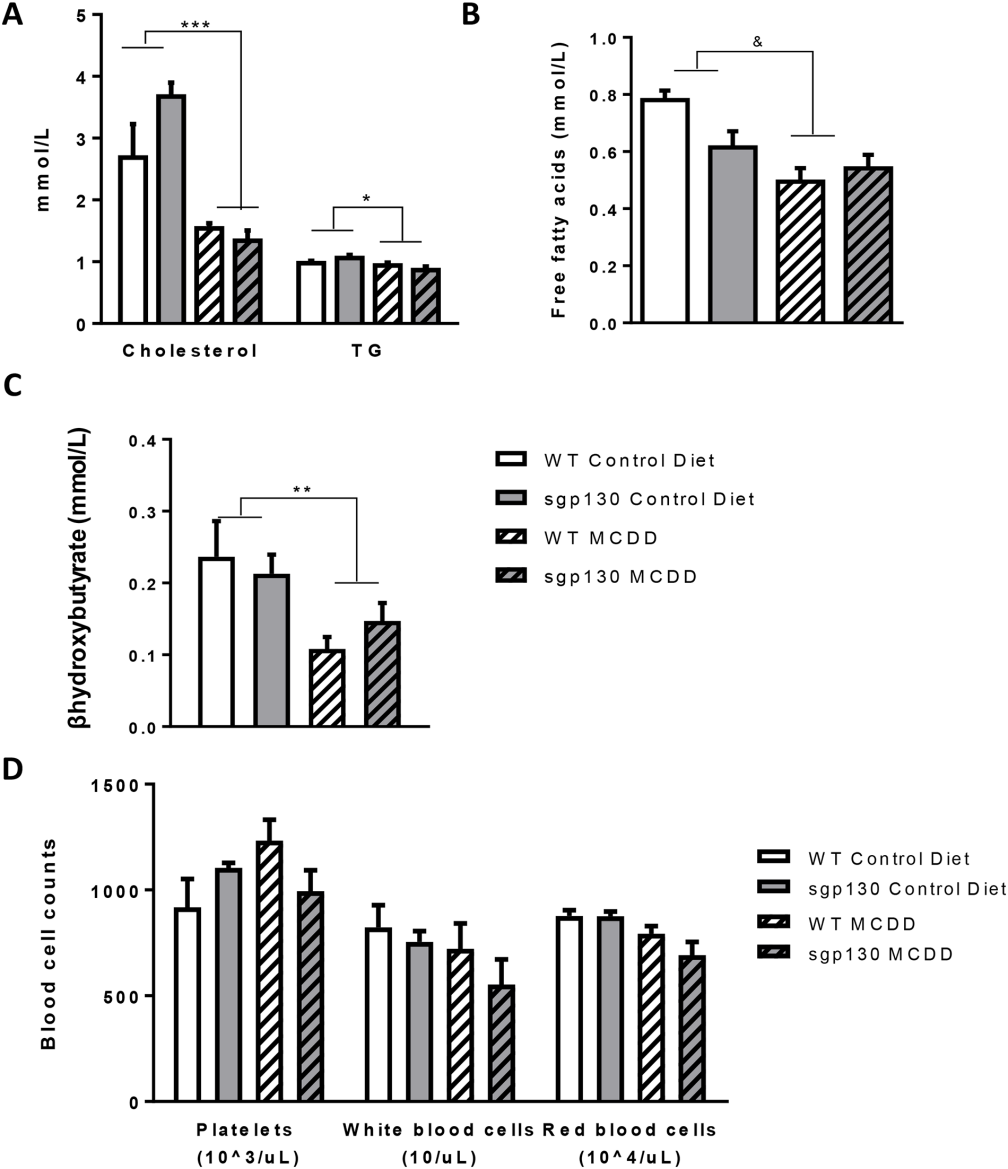
Overexpression of sgp130Fc does not prevent MCD diet-induced dyslipidemia. (A) Cholesterol and triglycerides, (B) free fatty acids and (C) βhydroxybutyrate concentrations (mmol/L) were measured in plasma of control and methionine and choline deficient diet (MCDD) mice after 4 weeks of diet intervention. Data expressed as average ± SEM, n = 6–7. Statistical analysis by 2 way ANOVA, **p<0.01; ***p<0.001 Main effect for Control vs MCDD; & p<0.05 Interaction for diet/genotype.

The MCD diet fed animals exhibited a reduction in circulating cholesterol, triglycerides and fatty acids (Fig 2A and 2B) consistent with an inhibition of hepatic lipoproteins secretion. In addition, we measured circulating β-hydroxybutyrate, a marker of β-oxidation, which was decreased in the MCD diet fed animals (Fig 2C). This was a surprising result as the MCD diet typically increases β-hydroxybutyrate [19]. The MCD diet did not affect circulating platelet, white or red blood cell counts (Fig 2D). None of these blood markers were affected by sgp130Fc irrespective of diet (Fig 2A–2D).

### Sgp130Fc transgenic mice develop a similar NASH pathology to wild type mice

**Fig 3:**
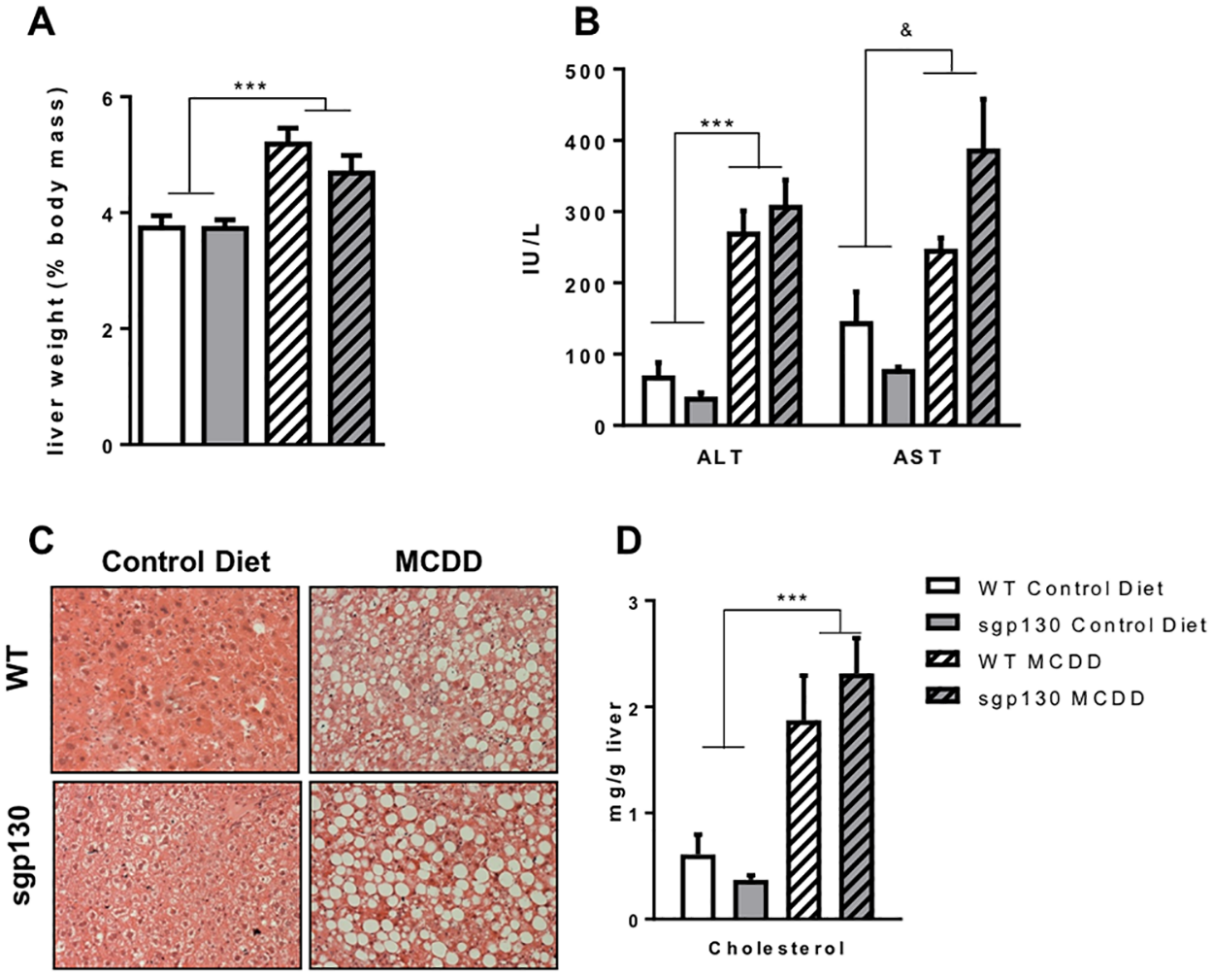
Sgp130Fc transgenic mice develop a similar NASH pathology to wild type mice on a MCD diet. Liver condition in 4 weeks fed control and methionine and choline deficient diet (MCDD) mice was assessed by studying (A) liver weight (expressed as % body weight), (B) liver enzymes ALT and AST, (C) liver histology with H&E staining and (D) intra-hepatic cholesterol concentrations. Data expressed as average ± SEM, n = 6–7. Statistical analysis by 2 way ANOVA, ***p<0.001 Main effect for Control vs MCDD; & p<0.05 for diet/genotype interaction.

The MCD diet induced hepatomegaly (Fig 3A) and liver injury as measured by circulating AST and ALT (Fig 3B). ALT in particular, considered the most relevant biomarker for liver health, was markedly increased (~5 fold) in the MCD diet fed animals. As expected, hepatosteatosis, as determined by haematoxylin and eosin staining of liver sections (Fig 3C), and liver cholesterol levels (Fig 3D) were markedly elevated in mice fed the MCD diet. None of these hallmarks of NASH were different when comparing mouse strains (Fig 3A–3D).

### Overexpression of sgp130Fc does not prevent MCD diet induced hepatic inflammation

**Fig 4:**
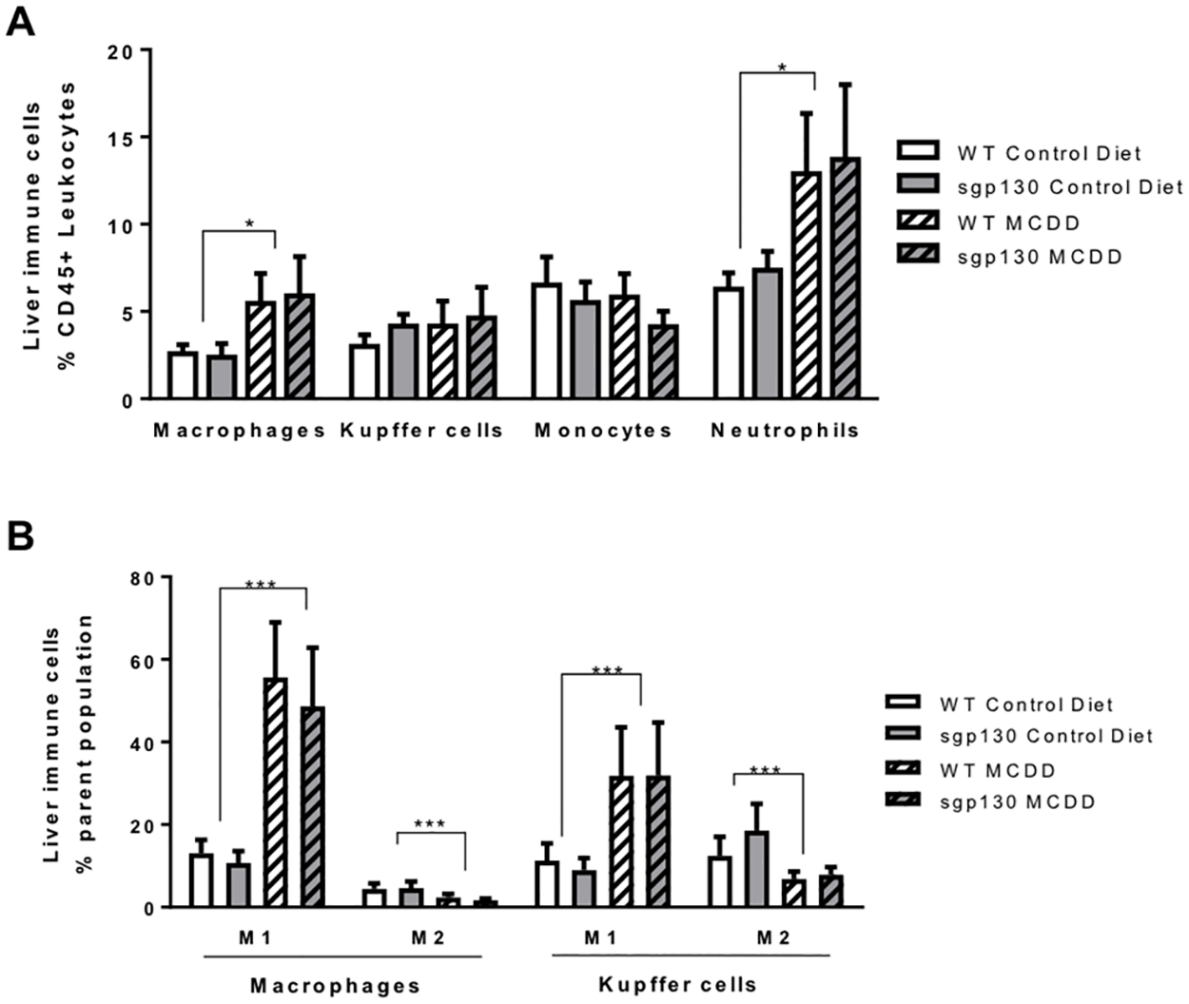
Sgp130Fc transgenic mice exhibit a similar inflammatory phenotype in response to a MCD diet. Flow cytometry on livers from 4 weeks fed control and methionine and choline deficient diet (MCDD) mice was performed and showed (A) liver immune cells liver as percent of leukocytes (CD45+ cells) and (B) macrophages and Kuppfer cells inflammatory phenotype as percent of total macrophages and Kuppfer cells respectively. Data expressed as average ± SEM, n = 6–7. Statistical analysis by 2 way ANOVA, * p<0.05; ***p<0.001 Main effect for Control vs MCDD.

We next carefully measured inflammatory markers in the livers of all mice. As expected, the MCD diet increased macrophage and neutrophil infiltration into the liver, however, monocytes and liver resident Kupffer cells numbers were unchanged compared with the control diet treated animals (Fig 4A). Surprisingly, we observe no change in the number of Kupffer cells in the liver of the animals receiving a MCD diet. Accordingly, we analysed the hepatic ratio of anti to pro-inflammatory macrophage and Kupffer cells. Macrophages and Kupffer cells are able to dynamically express a range of extra-cellular markers according to their environment. They are usually schematically presented as pro-inflammatory M1 or classically activated macrophages, when presenting markers such as the integrin CD11c or anti-inflammatory M2 or alternatively activated macrophages when harbouring markers such as the mannose receptor CD206 [20]. Using these antibodies, we observed a marked increase in M1, and a parallel decrease in the M2, myeloid cells in both macrophages and Kupffer cells (Fig 4B), consistent with NASH induced liver inflammation in MCD fed animals. However, contrary to our hypothesis, none of the immune cells populations were different when comparing genotypes (Fig 4A and 4B).

### MCD diet- induces NASH independently of the IL-6 pathway

**Fig 5:**
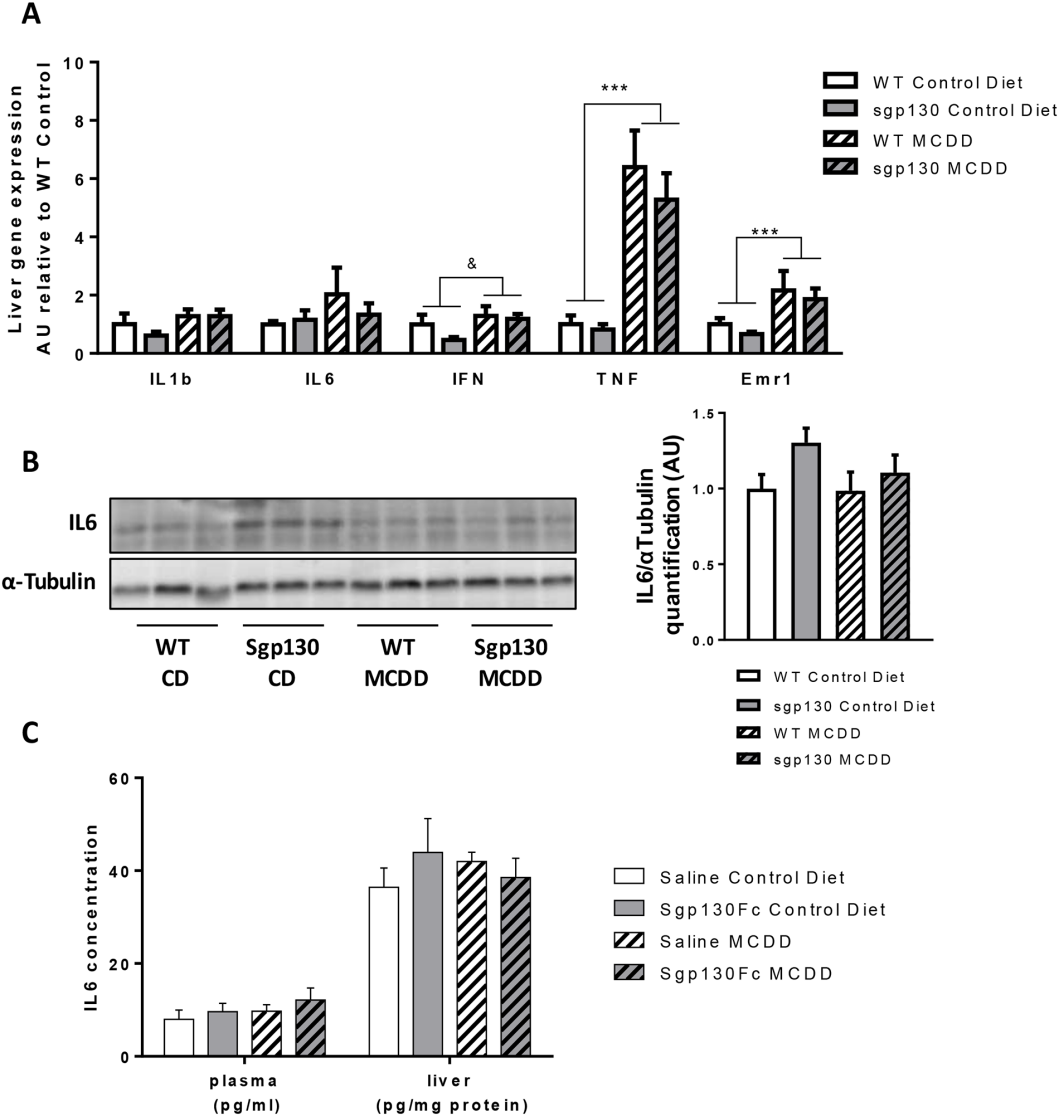
MCD diet induces NASH independently of IL-6 signalling. Liver samples from 4 weeks-fed control diet (CD) or methionine and choline deficient diet (MCDD) mice were homogenised and (A) mRNA expression of pro-inflammatory genes and macrophage marker was assessed by real time PCR and (B) protein expression of IL-6 and the loading control αTubulin was measured by Western blotting in the WT or transgenic animals. (C) Plasma and hepatic IL6 levels were measured in C57BL/6 fed a CD or MCDD for 4 weeks and injected with vehicle or sgp130Fc every second day. Data expressed as average ± SEM, n = 6–7. Statistical analysis by 2 way ANOVA, * p<0.05; **p<0.01; ***p<0.001 Main effect for Control vs MCDD; & p<0.05 for diet/genotype interaction.

Elevated IL-6 is an independent prognostic indicator of human NASH [5] and is a hallmark of mouse models of NASH [2]. Given these observations, along with our own previous findings that sgp130Fc overexpression blocks IL-6 induced inflammation [13, 16] we were, therefore, surprised by our observations that the sgp130Fc Tg mice were completely unprotected from the deleterious effects of the MCD diet. Despite previous evidence that the MCD diet induced increased IL-6 expression [8], we observed no increase in either IL-6 mRNA (Fig 5A) or protein (Fig 5B and 5C) expression in the liver as a result of the MCD diet, in all animals. In addition, we were unable to detect any changes in circulating or liver IL-6 concentrations in any of the animals fed a MCD diet and injected with saline or sgp130Fc (Fig 5C). We did, however, detect increased liver gene expression of TNF-α, IFN-γ and EMR1 (the gene encoding F4/80) in MCD diet fed mice (Fig 5A), suggesting that the MCD diet induces liver inflammation, but not via enhanced IL-6 expression. Given the lack of an effect of the MCD diet on IL-6 expression, it is not surprising that we did not see any effect of sgp130Fc overexpression or treatment on the MCD diet induced liver phenotype.

## Discussion

IL-6 is a main mediator of liver pathophysiology. Indeed, IL-6 has long been known to promote the synthesis of hepatic acute phase proteins [21]. These proteins, such as C-reactive protein play a strong role in inflammation resolution making them critical for fighting infections but they can also lead to states of chronic inflammation when increased. More importantly, with respect to the NASH pathology and its evolution, IL-6 is described as a central player in HCC. IL-6 serum levels are known to increase in patients with HCC and to correlate with the risk of developing HCC [22, 23].

The exact role of IL-6 in the pathogenesis of NASH is still not fully elucidated. However, both mouse models of NASH and patients show elevated IL-6 correlating with disease development and progression. Since IL-6 can have both detrimental and beneficial effects on the liver, using it as a therapeutical target could be a double edge sword. Indeed, both IL-6 overexpression and IL-6 KO mouse models present with liver inflammation, highlighting how balanced the signaling of this cytokine needs to be [9, 24]. In the last decade, we have demonstrated that much of the pro-inflammatory effects of IL-6 are transduced by the trans-signaling pathway, while classical IL-6 signaling in the liver appears to be anti-inflammatory [9, 14, 16]. Given the link between NASH, inflammation and IL-6, we reasoned it prudent to target the IL-6 pro-inflammatory pathway in a mouse model of NASH.

Several papers have previously reported a role for IL-6 in mouse models using the MCD diet to induce NASH. Yamaguchi and colleagues demonstrated a marked increase of the circulating levels of IL-6 in the MCD diet group. Furthermore, when they inhibited IL-6 signaling using the IL-6R antibody MR16-1, they observed decreased plasma ALT and lowered hepatic apoptosis [8]. Others have demonstrated that IL-6 KO animals exhibit a reduced liver inflammation and NASH score associated with decreased plasma ALT and an altered hepatic lipid profile [6]. Interestingly, in these two studies both IL-6 classical signaling and trans-signaling pathways were inhibited, meaning that the anti-inflammatory effects of IL-6 were also abolished. Given the previous results, we were expecting to see a clear improvement of the liver pathology in animals fed the MCD diet with over-expression of the IL-6 trans-signaling inhibitor, sgp130Fc. However, contrary to these previous findings, we observed no effect of the MCD diet on IL-6 expression measured in several different ways (Fig 5). It was unsurprising, therefore, that blocking IL-6 trans-signaling had no effect on the liver pathogenesis of MCD diet fed mice. There are some important methodological differences when comparing previous studies with the data reported herein. Mas et al used a global knock out model to ablate IL-6 signalling and studied the mice on a MCD diet for 5 weeks, making the model similar to the one we used [6]. However, the congenital deficiency of all IL-6 signaling could lead to some biological adaptations that could confer some protection during the development of the MCD diet-induced NASH phenotype. Of note, these authors did not present data regarding the circulating levels of IL-6 in the WT controls, making it impossible to acknowledge the direct role of the cytokine in this mouse model. In contrast with our data, Yamaguchi and colleagues reported a markedly increased plasma IL-6 concentration in mice fed a MCD diet. Importantly, however, they report these IL-6 levels in mice that were fed a MCD diet for 8 weeks, twice as long as reported herein, and their mice continuously lost weight over the course of the diet intervention. Their studies were performed, therefore, in 14 week old mice weighing an average of 12 grams [8], rendering this a mouse model of cachexia, which had the potential to complicate the role of IL-6 in the development of MCD diet-induced NASH development.

In our study, we report that TNF-α was increased (Fig 5A). TNFα is known to be a key pro-inflammatory marker, contributing to the chronic low-grade inflammatory state observed in human with metabolic disease [25]. In addition, TNF-α has been implicated in the pathogenesis of NASH in both humans and rodent models [26–29]. Of interest for this study, several groups have reported the implication of TNF-α, originating from inflamed Kupffer cells in the MCD diet-induced NASH mouse model [27, 29]. In accordance with these publications, we report an increased expression of TNFα gene expression as well as a switch in the inflammatory phenotype of the Kupffer cells. Consistent with these previous studies, our results suggest that in the mice receiving a MCD diet, the hepatic cholesterol and lipid accumulation triggers the switch towards the pro-inflammatory phenotype in the resident macrophages as well as recruitment of circulating monocytes, which all contribute to TNF-α production and enhanced liver inflammation and injury.

Our results indicate that the MCD diet leads to a NASH associated pathogenesis in mice, which while sharing some similarities with human NASH, is largely a different pro-inflammatory profile. It is likely that this is associated with the major caveat of the MCD diet model—that being the marked weight loss observed in animals on this diet. All rodents on a MCD diet exhibit a 20 to 30% body weight loss in the first 4 weeks of that diet which is obviously a very different feature to what is documented in human NASH patients. A strong proportion of NASH patients present a BMI over 25 being either overweight or obese and conversely obese patients have an increased risk of developing NAFLD and NASH [1]. One can hypothesize that this increased adiposity plays an important role in the development of the pathology as the fat tissue is a central endocrine regulator known to secrete many cytokines and to be dysregulated during obesity [30]. Indeed, in obesity, the circulating levels of IL-6 correlate with adiposity and IL-6 expression increases in the adipose of obese patients [31–33]. Hence, the IL-6 component observed in human NASH, as well as some rodent models could arise from an adipose tissue—liver axis, which has been previously described as central in metabolic disorders but is also emerging in alcoholic liver disease [34]. The loss of adipose tissue (down to 1g of fat mass in the MCD diet-fed WT animals vs more than 6g for the control WT) likely explains the absence of IL-6 phenotype in our MCD diet-fed mice and the lack of effect of sgp130Fc over-expression.

In conclusion, our results suggest that the MCD diet should be avoided as a model of NASH due to its inability to phenocopy the inflammatory profile of human NASH of which elevated IL-6 is a hallmark of the NASH pathogenesis.

## Methods

### Mouse models

Sgp130Fc Tg mice were generated as previously described [14, 16]. Briefly, heterozygous animals were bred together and litters were genotyped. Animals negative for the transgene were designated Wild Type (WT) and animals expressing the transgene were bled for ELISA analysis as genotyping cannot distinguish between homozygous and heterozygous mice. Mice exhibiting a human sgp130Fc circulating concentration of more than 10μg/ml were considered homozygous while animals testing positive, but below that threshold, were considered as heterozygous. Littermate WT and homozygous mice were bred in parallel for no more than one generation and their litters were used for the study. Sgp130Fc Tg, WT and a separate cohort of C57BL/6 mice were bred and housed at the Alfred Medical Research and Education Precinct Animal Centre in a pathogen free facility under controlled environmental conditions and exposed to a 12:12 h light:dark cycle. For the injection study, C57BL/6 mice were injected intra-peritoneally every second day with 0.5mg/kg sgp130Fc. Animal experiments were approved by the Alfred Medical Research and Education Precinct Animal Ethics Committee and conducted in accordance with the National Health and Medical Research Council of Australia Guidelines for Animal Experimentation. All experiments commenced when mice were 10–12 weeks of age.

### Dietary intervention

Mice were fed a high fat (21% energy from fat) sucrose diet depleted in methionine and choline (methionine and choline deficient; MCD diet) or the same diet but with repletion of standard levels (according to the AIN93G formulation) of methionine and choline (control diet). Animals were provided with food and water *ad libitum*.

### Metabolic measurements

#### Plasma biochemistry measurements

Concentrations of the enzymes aspartate transaminase (AST) and alanine transaminase (ALT) as well as cholesterol, triglycerides, bile acids and β-hydroxybutyrate were measured by ASAP laboratories, Melbourne, Australia using a Dade Behring Dimension^®^ Xpand^®^ Integrated Chemistry System. Non-esterified fatty acids (NEFA) were measured using the WAKO NEFA kit (Wako Chemicals, Japan) according to manufacturer’s instructions.

#### Body composition

Mouse body composition (fat mass (FM) and lean body mass (LBM)) was measured weekly with a 4-in-1 EchoMRI body composition analyzer (Columbus Instruments, USA) and standard laboratory scales.

### Histology

Liver was fixed in 4% paraformaldehyde before being stained with Mayer’s Haemotoxylin and Eosin for histological determination of tissue structure. Stained sections were imaged microscopically (Olympus BX50) and captured using computer software (QCapture Pro 6.0).

### RNA extraction and real time quantitative PCR

RNA extraction was performed as previously described [16]. Briefly total RNA was isolated from tissues with Tri Reagent^®^ (Sigma Aldrich) and reverse transcribed to cDNA with the use of random hexamers. Gene expression analysis was performed by Real-time PCR on a 7500 fast sequence detector (Applied Biosystems) using TaqMan gene expression assays (Applied Biosystems), including an 18S probe and primers for housekeeping measurements.

### Western blotting & ELISA assay

Tissue samples were lysed and protein concentration determined as previously described [35]. Immunoblotting was performed using the following primary antibodies: IL-6 & αTubulin (Cell Signaling Technology, USA). IL-6 was detected in plasma and liver homogenate using an IL-6 quantikine ELISA (R&D systems, USA). Liver was homogenised in RIPA buffer before protein assay and a 1mg/ml solution prepared for ELISA assessment.

### Blood parameters

For haematological assessment, a small volume (20 μL) of whole blood was diluted 1:7 in Sysmex CELLPACK^™^ (Sysmex, Japan) diluent and assessed using an automated haematology analyser (Sysmex XS-1000*i*, Japan).

### Flow cytometry

Immune cells were isolated from liver samples. Briefly, liver was finely diced and digested in collagenase B (Roche). Homogenate was spun and filtered and the pellet obtained was resuspended in red blood cell lysis buffer before being washed and used for flow cytometry. Fluorochrome-conjugated antibodies against the following antigens were used for flow cytometry: CD45 (30-F11), F4/80 (BM8), CD11c (N418), CD206 (MR5D3) (eBiosciences) and CD11b (M170), NK1.1 (PK136) (BD Biosciences).

### Statistical analysis

Data are presented as mean ± standard error of the mean (SEM). Two-way analysis of variance (ANOVA) was used to detect main effects for diet (chow vs. HFD) and genotype (WT vs. sgp130Fc), Post-hoc analyses (Holm-Sidak) were performed when a significant interaction effect occurred. Where else reported, one-way ANOVA or a student T-Test was used. Analyses were performed using a statistical computer program (Sigma Stat Version 3.5). p<0.05 was considered statistically significant.
